# A Case Presentation of a Patient with Microsatellite Instability and BRAF Mutant Metastatic Colon Cancer and Bibliography Update

**DOI:** 10.1155/2019/4767019

**Published:** 2019-02-17

**Authors:** K. Thomopoulou, M. Tzardi, D. Mavroudis, I. Souglakos

**Affiliations:** ^1^Department of Medical Oncology, University General Hospital of Heraklion, Heraklion, Greece; ^2^Laboratory Department of Pathology, Medical School, University of Crete, Greece

## Abstract

This is a case of a patient who presented to the emergency department with acute abdominal pain due to bowel obstruction. An extended right hemicolectomy with ileosigmoid anastomosis due to an obstructing mass on the splenic flexure was urgently performed. During operation, liver and peritoneal lesions were detected and samples were also sent for histological analysis. Pathology report was consistent with poorly differentiated mucinous adenocarcinoma with signet ring cells; peritoneal lesions were confirmed histologically as metastatic. Genetic testing revealed the BRAF^V600E^ mutation and mismatch repair deficiency (dMMR). After progressing on 1st line chemotherapy, the patient has a continuing and long-lasting partial response to 2nd line treatment with pembrolizumab.

## 1. Introduction

Colorectal cancer (CRC) is the third most common cancer in men and the second in women accounting approximately 1.6 million new cases and 830,000 deaths each year [1]. CRC is a very heterogenic disease and molecular characterization based on mismatch repair (MMR), BRAF/RAS mutation status, which has a significant prognostic and predictive value, has become mandatory for daily clinical practice, since it may alter treatment strategy [2].

The development of CRC involves genetic and epigenetic alterations that can lead to proliferation, apoptosis, and angiogenesis. One of the better characterized pathways is that of *BRAF* activating mutations, which lead to MAPK constant activation. *BRAF* mutation, more common at codon 600 (V600E), can activate the mitogen-activated protein kinase (MAPK) signalling leading to tumourigenesis [3]. *BRAF^V600E^* is found approximately in 8-10% of CRC cases and is associated with significantly lower median overall survival [4, 5]. In contrast, recent data indicate that other non-600E *BRAF* mutations occur in 2.2% of mCRC and are associated with better prognosis [6].

Furthermore, *BRAF^V600E^* mutation is associated with cyclin D1 activation and microsatellite instability (MSI-H) [5, 7], increased age, performance status 2, and peritoneal metastasis [5]. When this mutation is present and there is microsatellite stability (MS-S), it has a negative prognostic value with poor survival [4, 8–11].

MSI high phenotype is created by the loss of function of four mismatch repair genes (MMR) that are responsible for correcting single base pair mismatches [12]. Germline loss of the MMR system leads to Lynch syndrome, whereas somatic mutations are present in about 10% of sporadic colon cancer patients [13]. MSI-H tumours are considered to have better prognosis compared to MS-S cancers. MSI-H cancers harbouring the *BRAF^V600E^* mutation usually have deficient MMR (dMMR) through the hypermethylation of the MLH1 gene and the CpG island methylator phenotype (CIMP+ pathway) and are exclusively sporadic [14]. Patients with simultaneous detection of *BRAF^V600E^* mutation and dMMR in their tumours have better prognosis in comparison with those with *BRAF^V600E^* mutation and proficient MMR (pMMR) [15, 16].

In recent years, alternative treatment options are emerging including immune checkpoint inhibitors. More specifically, programmed cell death protein 1 (PD1) is a protein on the surface of lymphocytes which binds to PDL1/PDL2 protein located on the surface of cancer cells. When bound, it leads to the suppression of inflammatory activity via the downregulation of T-effector cells and the upregulation of T-regulatory cells [17]. Pembrolizumab is a humanised IG4 monoclonal antibody which blocks the PD1 protein, leading to the activation of immune response against tumour cells. Blocking this pathway has led to spectacular responses in other immunogenic tumours such as melanoma and lung cancer [18]. In colon cancer, dMMR tumours are associated with high lymphocytic infiltration in the tumour microenvironment, which translates in good responses to immune checkpoint inhibitors. This is also supported by a recent phase II study by Le et al. showing that response to PD1 inhibitors could be predicted by evaluating the MSI status [19].

Here, we present a case of a patient with de novo metastatic *BRAF^V600E^* mutated and dMMR mCRC who has a continuing and long-lasting partial response to 2nd line treatment with pembrolizumab.

## 2. Case Presentation

A 66-year-old female with a past medical history of hypertension and absent family history of cancer presented to the emergency department with acute abdominal pain due to bowel obstruction in July 2016. Her symptoms had started about a year before when she had periodically noticed a change in bowel movements and an increasing palpable mass in the left abdomen.

**Figure 1:**
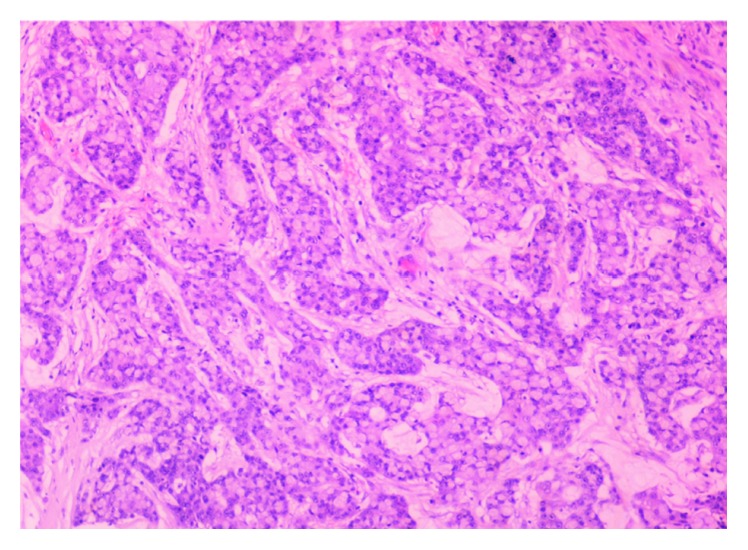
Hematoxylin and eosin staining (original magnification ×400): poorly differentiated with signet ring colon adenocarcinoma with extracellular mucin.

**Figure 2:**
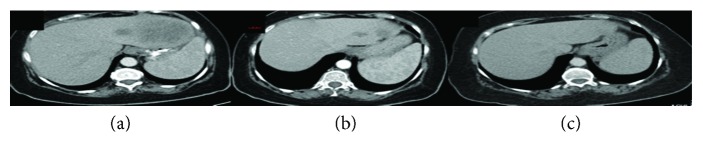
CT scan that shows liver lesions (a) before starting 1st line chemotherapy and (b) before starting immunotherapy. (c) Last CT scan showing continuous PR.

An extended right hemicolectomy with ileosigmoid anastomosis due to an obstructing mass on the splenic flexure was urgently performed. During operation, liver and peritoneal lesions were detected and samples were also sent for histological analysis. Pathology report was consistent with poorly differentiated mucinous adenocarcinoma with signet ring cells (Figure 1), pT4N2bM1, with 14 positive lymph nodes out of the 40 retrieved. The liver and peritoneal lesions were confirmed histologically as metastatic. Genetic testing by Ion Torrent NGS system revealed the *BRAF^V600E^* mutation, loss of function mutation of *LKB1*, and mismatch repair deficiency (dMMR), and at that time, it was felt that these genetic alterations were consistent with a sporadic colon tumour. Immunohistochemistry for PDL1 was not performed, since it does not have predictive value in dMMR tumours. CT of the chest/abdomen and pelvis (CAP) showed multiple enlarged abdominal lymph nodes, at least seven liver lesions (Figure 2(a)), metastasis to the left adrenal gland, multiple peritoneal metastases, and a block of supraclavicular lymph nodes measuring 1.9 cm.

At that time, she had a performance status (PS) 1 and had fully recovered from surgery. After a very thorough discussion about treatment options, the patient was elected to participate in the open-label phase II MINOAS trial (NCT02624726), which is aimed at studying the combination of FOLFIRI regimen plus aflibercept in the 1st line setting in metastatic colorectal cancer. In October 2016, the patient was started on chemotherapy with FOLFIRI consisted of day 1, 5-fluorouracil push (400 mg/m^2^); day 1 and 2, 5-FU continuous infusion (1200 mg/m^2^); and day 1 leucovorin (400 mg/m^2^) and irinotecan (180 mg/m^2^) combined with aflibercept at a dose of 4 mg/kg repeated every 2 weeks. She had a major clinical benefit; however, she developed grade IV neutropenia which led to 15% dose reduction of 5-FU and CPT regimen. She was evaluated by CT CAP at 3 and 6 months of treatment, which showed partial response (PR), and it was then decided to continue with maintenance therapy of aflibercept biweekly. She remained in maintenance therapy for 2 months; when she started losing weight, she had loss of appetite and abdominal aches. CT CAP revealed progression of disease (PD) (Figure 2(b)) with increasing abdominal lymph nodes and peritoneal metastases by more than 30% (based on RECIST criteria).

On August 2017, based on the fact that she had PD and her disease was MMR deficient, the patient started 2nd line treatment with pembrolizumab at a fix dose of 200 mg every 3 weeks. She had evaluation of her disease with CT CAP every 8 weeks to assess response to treatment. Within the first 4 weeks, the abdominal pain disappeared and she gained weight (12 kg). At week 8, she had achieved a partial response with decreasing liver lesions, abdominal lymph nodes, and peritoneal masses. Her CT scans after 16 weeks showed continuous PR. She has been tolerating immunotherapy well and developed only grade I arthralgia and diarrhea that improved with paracetamol and antidiarrheal drugs. She is still continuing pembrolizumab every 3 weeks and her most recent CT scans from late September 2018 showed further decrease in liver lesions (Figure 2(c)) and supraclavicular lymph nodes measuring 7 mm (1.9 cm at the start of treatment) and decrease by more than 20% in abdominal lymph nodes while peritoneal masses have totally disappeared.

## 3. Discussion

We describe the case of MSI-H, *BRAF^V600E^* mutated, and dMMR CRC, which was de novo metastatic to abdominal and supraclavicular lymph nodes with extensive liver disease and who is still on a continuous partial response since starting 2nd line treatment with immunotherapy.

Firstly, here we present the case of a 66-year-old lady who had a T4 tumour and a high volume of lymph node disease and peritoneal dissemination from the time of diagnosis. Searching the literature, Kang et al. suggested in a retrospective study that MSI-H tumours often have a high lymph node load and are usually poorly differentiated T4 tumours with better overall survival and a different metastatic pattern compared to MSI-L BRAF mutant tumours [20]. Also, in a previously published study from our group, in the biggest prospective cohort of patients with *BRAF^V600E^* mutation, they tend to be older, have a PS of 2, and more frequently present peritoneal metastases [5]. Furthermore, in our case, the primary tumour was located in the distal colon. Several studies reported that MSI-H and BRAF mutant tumours are commonly observed in the proximal colon, where molecular identification exhibits more often the BRAF mutation and CpG island methylation and are associated with poorer prognosis [21–25]. Therefore, in our case, MSI-H in the distal colon might be one of the factors that determined a better prognosis, despite baring the BRAF mutation.

Moreover, the patient responded well to 1st line chemotherapy with FOLFIRI aflibercept reaching a PFS of 9 months. Apparently, this does not come as a surprise since multiple studies have shown that MSI-H colon cancer cells are more sensitive to doublet with irinotecan, which is a topoisomerase inhibitor, compared to a doublet with oxaliplatin. In a recent trial, patients with MSI-H CRC had an overall complete response rate more than 60% to neoadjuvant irinotecan compared to only 20% in MSS CRC [26]. However, other retrospective analyses suggested that the MSI status cannot definitely predict the response to a specific type of chemotherapy [27]. Furthermore, a subgroup analysis of the VELOUR trial has reported statistically significant benefit for the addition of aflibercept to FOLFIRI as 2nd line treatment of mCRC, in patients *BRAF^V600E^* mutation [28].

Furthermore, in this case, we observe that our patient has been having continuous response to anti-PD1 treatment. Two main clinical studies uncovered the activity of PD-1 inhibitor in metastatic MSI-H CRC. The first phase II study, Keynote 164, studied the activity of pembrolizumab in 2nd and 3rd line setting in MSI-H and MSS CRC patients. Forty-one patients were enrolled and received intravenously pembrolizumab at a dose of 10 mg/kg biweekly. Patients were divided in 3 subgroups: the MMR-D (*n* = 11), the MMR-P (*n* = 21), with 1 patient being *BRAF* mutant and dMMR of noncolon cancer. Median 20-week PFS and immune-related overall response rate (ORR) for MSI-H patients were 78% and 40%, respectively, whereas for MSS CRC it was 11% and 0%. Median PFS and OS for MMR-P patients were 2.2 and 5 months and not reached for MSI-H CRC. Main adverse events included fatigue (32%), diarrhea (24%), pruritus (24%), and hematologic toxicity. This trial however included only one patient with MSS and BRAF mutant CRC, and as a result, conclusions could not be drawn about response to immunotherapy in patients bearing both conditions [19].

Overman et al. conducted a multicentre open-label phase II trial, the Checkmate 142 trial, evaluating the activity of nivolumab in MSI-H CRC. Seventy-four patients, MSI-H patients, received nivolumab 3 mg/kg every 2 weeks, of which 12 were BRAF mutant. ORR was 27% and stable disease showed 37.8%. 12-month PFS and OS were 48.9% and 73.8%, respectively. Grade 3/4 immune-related adverse events were observed in 20% with more common elevated lipase and amylase. Interestingly, 25% of patients with BRAF mutant disease achieved an objective response, and 75% disease control at 12 weeks, overcoming the known poor objective responses with chemotherapy (less than 10%) or with inhibition with BRAF, EGFR, and MEK (approximately 15%) [29]. The same study group presented at ESMO Congress 2018 the updated results of the combination of nivolumab (3 mg/kg given biweekly) and low-dose ipilimumab (1 mg/kg given every 6 weeks) on the 1st line setting in 45 patients with d-MMR metastatic colorectal cancer. Of these, 17 patients carried the BRAF mutation. Notably, ORR reached 60%, whereas 12-month PFS and OS were 77% and 83%, respectively [30].

Until recently, there were many trials that failed to prove any efficacy of single-agent BRAF inhibition in patients with BRAF mutant CRC [31, 32]. A proven explanation is that the blockade of BRAF leads to feedback increase of EGFR activation and consequently reactivation of the MAPK pathway [33, 34]. Based on that, there are a few trials assessing the efficacy of BRAF/MEK inhibition combined with chemotherapy in patients with BRAF mutation. The first randomised controlled phase II SWOG 1406 study was conducted by the Southwest Oncology Group, which evaluated the addition of vemurafenib (960 mg PO twice daily) in combination with irinotecan (180 mg/m^2^ IV every 14 days) plus cetuximab (500 mg/kg every 14 days). One hundred and six BRAF mut/RAS wt patients were enrolled who had received one or more prior chemotherapies, including 54 on the arm with the triple treatment. Median PFS for the triplet and doublet treatments was 4.4 versus 2.0 months, respectively, and response rate achieved was 16% on the triplet arm compared to 4% on the doublet. Of note, anaemia and neutropenia were more frequent on the vemurafenib arm; however, other treatment-related AE were comparable in both subgroups. Interestingly, 13 patients who were confirmed to have MSI-H tumours benefited from triplet combination (HR: 0.50, 95% CI: 01-1.6) [35].

The BEACON trial is an ongoing randomised phase III trial including patients bearing the BRAF^V600E^ mutation and who had received at least one line of chemotherapy, randomized 1 : 1 : 1 to receive encorafenib (BRAF inhibitor) plus binimetinib (MEK inhibitor) and cetuximab (A arm) versus encorafenib plus cetuximab (B arm) versus irinotecan-based chemotherapy combined with cetuximab (control arm). The first safety results of the combination of arm A were released at the ESMO gastrointestinal congress in June 2018. Median PFS was 8 months and 12-month OS for this group of patients reached 62%. Moreover, ORR was 48% compared to 62% in patients who had received only one line of chemotherapy. Lastly, adverse events were similar to the other trials and more common AE were fatigue (13%), anaemia (10%), and elevated liver enzymes (10%) [36].

## 4. Conclusion

In conclusion, we present the case of a patient with MMR-D BRAF mutant metastatic CRC who is still responding to immunotherapy, and if progression of disease occurs, she could receive targeted inhibition with BRAF/MEK/anti-EGFR or irinotecan/anti-EGFR and vemurafenib. CRC is historically a disease that harbours biomarkers that could predict response to treatment and targeted therapies. *BRAF* mutant colon cancer came as an exception to that rule. However, despite the fact that *BRAF^V600E^* mutation pMMR CRC bears worse survival than *BRAF^V600E^* mutation dMMR, fortunately, many clinical trials emerged, and a step forward has been made to understand better the biologic behaviour of this disease. Many questions still need to be answered though. What is the ideal sequence of treatments? All trials exploring the efficacy of immunotherapy and *BRAF* inhibition included patients who had received at least one line of chemotherapy. Furthermore, to the best of our knowledge, there is no trial comparing immunotherapy versus triplet inhibition. Looking to the future, we are optimistic that new clinical data will provide more information for the optimal treatment of this particular subgroup of patients.
